# Exploration of the TRIM Fold of MuRF1 Using EPR Reveals a Canonical Antiparallel Structure and Extended COS-Box

**DOI:** 10.1016/j.jmb.2019.05.025

**Published:** 2019-07-12

**Authors:** Michael Stevens, Barbara Franke, Katarzyna A. Skorupka, David S. Cafiso, Owen Pornillos, Olga Mayans, David G. Norman

**Affiliations:** 1 Nucleic Acids Structure Research Group, University of Dundee, Dundee, United Kingdom; 2 Department of Biology, University of Konstanz, 78457 Konstanz, Germany; 3 Department of Molecular Physiology and Biological Physics, University of Virginia, 22908 Charlottesville, VA, USA; 4 Department of Chemistry, University of Virginia, Charlottesville, Virginia, USA

**Keywords:** TRIM fold, MuRF1, coiled-coil, PELDOR, disulfide cross-linking, HD, helical domain, MuRF, muscle-specific RING finger, EPR, electron paramagnetic resonance, PELDOR, pulsed electron–electron double resonance, MTSSL, (1-oxyl-2,2,5,5-tetramethylpyrroline-3-methyl) methanethiosulfonate, AT, acidic tail

## Abstract

MuRF1 (TRIM63) is a RING-type E3 ubiquitin ligase with a predicted tripartite TRIM fold. TRIM proteins rely upon the correct placement of an N-terminal RING domain, with respect to C-terminal, specific substrate-binding domains. The TRIM domain organization is orchestrated by a central helical domain that forms an antiparallel coiled-coil motif and mediates the dimerization of the fold. MuRF1 has a reduced TRIM composition characterized by a lack of specific substrate binding domains, but contains in its helical domain a conserved sequence motif termed COS-box that has been speculated to fold independently into an α-hairpin. These characteristics had led to question whether MuRF1 adopts a canonical TRIM fold. Using a combination of electron paramagnetic resonance, on spin-labeled protein, and disulfide crosslinking, we show that TRIM63 follows the structural conservation of the TRIM dimerization domain, observed in other proteins. We also show that the COS-box motif folds back onto the dimerization coiled-coil motif, predictably forming a four-helical bundle at the center of the protein and emulating the architecture of canonical TRIMs.

## Highlights

• The dimerization domain of MuRF1 (TRIM63) is an antiparallel coiled-coil.
• The COS-box motif folds back onto the center of the dimerization coiled-coil motif.
• Proteins in the classes CI and CII of the TRIM family exhibit a canonical TRIM fold.

## Graphical Abstract

**Figure:**
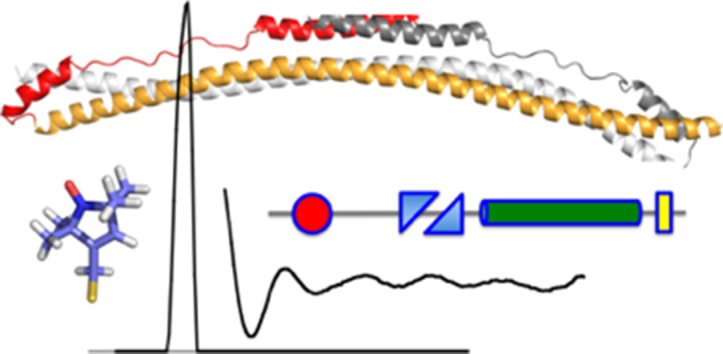

## Introduction

The TRIM protein family is the largest family of RING E3 ubiquitin ligases [1]. TRIM E3 ligases recruit E2-ubiquitin-conjugating enzymes to their respective substrates, thereby mediating the ubiquitination of proteins in the cell. Ubiquitination often leads to target turnover and promotes protein catabolism, but it can also serve numerous other functional roles such as regulation of the sub-cellular localization of target proteins or cell signaling. Not surprisingly, TRIM proteins have been associated with a variety of cellular processes, including activation of immune responses, regulation of gene expression, apoptosis and antiviral defense [2] [3]. They have also been linked to numerous pathologies including cancer, familiar Mediterranean fever, Opitz/BBB syndrome or nanism, among others [2] [4].

TRIM proteins are defined by their domain composition. Invariably, they share a tripartite fold consisting of a RING domain (R), one or two RING-like B-box domains (B), and a helical domain (HD) that forms a coiled-coil motif [5]. This N-terminal fold is commonly followed by one or more variable domains in C-terminal position (e.g., FnIII, PHD, B30.2). The variable domains are responsible for the binding of ubiquitination substrates and are specific to each TRIM class. To date, more than 70 human protein members of the TRIM family have been identified and indexed into 11 distinct classes (CI-CXI; *C* signifies *C*-terminal subgroup) according to their domain composition [4].

The ubiquitin ligase activity of TRIM proteins is reliant upon the placement of the RING domain with respect to the substrate-binding domain. There are currently no crystal structures of full-length TRIM proteins, but several structures of TRIM components are available that reveal the organization of the TRIM fold (reviewed in Ref. [1]). Of special relevance are the structures from TRIM5α-BHD (PDB ID 4TN3; [6]), TRIM20-HD-B30.2 (4CG4; [7]), TRIM25-HD (4LTB, 4CFG; [8]), TRIM25-HD-B30.2 (6FLN; [9]) and TRIM69-HD (4NQJ; [10]). These structures show that the HD component forms an obligate antiparallel dimer. The dimer comprises a long helix (H1) that forms an antiparallel coiled-coil, followed by a more flexible sequence containing two shorter helices (H2 and H3). This flexible sequence, which acts as a link to the C-terminal domains, packs against the antiparallel coiled-coil, predictably bringing the variable C-terminal domains in proximity to the N-terminal RING domains. The B-box domain acts as a coiled-coil capping feature, possibly providing stability to the helical scaffold.

**Figure 1:**
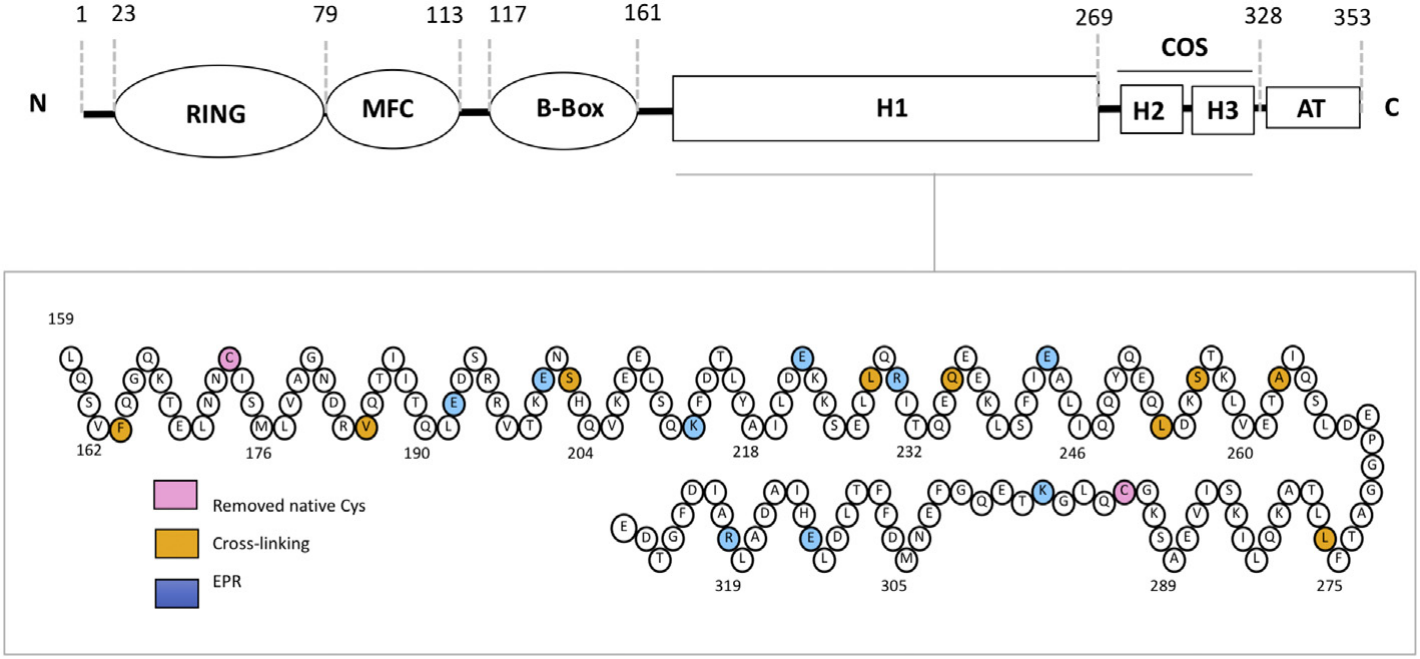
MuRF1 domain composition. MFC refers to a MuRF family-specific motif, and AT denotes a C-terminal acidic tail, which is predicted to be unstructured. Key sequence positions are indicated. The lower diagram shows the sequence composition of the HD of MuRF1 where the residue positions used in this study are indicated. Predicted helical portions are indicated by a zig-zag arrangement.

TRIM proteins in class C-II are unusual in that they lack variable C-terminal domains [3] (Fig. 1). In mammalians, this class consists of three highly conserved *Mu*scle-specific *R*ING *F*inger proteins (MuRFs): MuRF1, MuRF2 and MuRF3 that regulate the trophicity of striated muscle tissue. Because of its patho-physiological significance, MuRF1 is the best-studied member of the family. MuRF1 is strongly upregulated by atrophic stimuli, and it has been associated with the muscle atrophy that ensues upon immobilization, denervation, nutritional deprivation, aging or chronic disease [11]. MuRF1 deletion attenuates muscle wasting, and it is a pursued pharmacological target [12], [13], [14]. The structure of the B-box domain of MuRF1 [15] and MuRF2 (PDB ID 3Q1D) showed the domain to form dimers. A distinct feature of MuRFs is that the C-terminal fraction of their HD domain (spanning predicted helices H2–H3) contains a conserved sequence motif, termed the COS (*C*-terminal subgroup *O*ne *S*ignature)-box [16] (Fig. 1). This motif is also found in TRIM classes C-I and C-III, where it occurs just prior to a FnIII variable domain [16]. The structure of the isolated COS-box from MID1 has been elucidated using NMR [17]. It adopts a helical-hairpin fold that does not resemble the C-terminal region of the HD domain in other TRIM structures. The MID1-COS structure agrees with an earlier *ab initio* structure prediction for the COS-box of MuRF1 [18]. In both MID1 and MuRF1, the COS-box alpha-hairpin has been suggested to fold against the central H1 coiled-coil forming a minimal spectrin-like motif [18] [17]. Functionally, the COS-box is thought to mediate the association of its containing TRIMs to cytoskeletal structures. The COS-box of MID1 has been shown to associate with microtubules [16] [17], while the COS-box of MuRF1 was seen to interact with the sarcomere in transgenic mouse muscle [18]. Taken together, available data open the question of whether the COS-box constitutes a defined, protein-interaction, structural domain and whether the small TRIM fold of MuRF proteins might diverge from the canonical TRIM model.

The measurement of distances between nitroxide groups (introduced in proteins using site-specific labels) by pulsed electron paramagnetic resonance (EPR) is an established technique for the sampling of protein folds. This method often makes use of the pulsed electron–electron double resonance (PELDOR) protocol, also called double electron–electron resonance (DEER) [19]. PELDOR can measure distances of up to ~ 80 Å in most cases and can measure considerably longer distances in perdeuterated samples [20]. The distance measurements can be made with considerable accuracy (to within 1 Å depending on the quality of the data) and also describe the distance distribution, allowing regions of conformational flexibility to be defined. The attachment of nitroxide groups is commonly achieved by exploiting the reactivity of (1-oxyl-2,2,5,5-tetramethylpyrroline-3-methyl) methanethiosulfonate (MTSSL) [21] for the SH group of cysteine residues, which are introduced at desired positions in proteins by site-directed mutagenesis. The choice of position for the mutations is critical as replacement of any amino acids that are involved in tertiary interactions could alter the protein structure. The sites where labels are to be introduced also need to be solvent exposed to achieve quantitative labeling. Using a limited number of PELDOR-derived distance measurements does not allow for the detailed structural analysis of a protein fold. However, when the fold is known (e.g., if homologous structures exist), the approach can be used to explore conformational arrangements.

In this study, we employed disulfide cross-linking and the PELDOR experiment to investigate the quaternary structure of the MuRF1-HD dimer and the fold of its COS-box. Using site-directed mutagenesis, native cysteine residues were removed from the MuRF1-HD construct and new cysteine groups introduced at desired positions for labeling with MTSSL. Positions were chosen in this study guided by known TRIM structures taken from The Protein Data Bank [22]. The results show that helix H1 in MuRF1-HD forms a long antiparallel coiled-coil dimerization motif. We find no evidence for the folding of the COS-box into an alpha-hairpin motif. Instead, our data suggest that the COS-box is a semi-flexible extension that packs against the coiled-coil emulating the linker region that connects to C-terminal domains in other TRIM proteins. Thus, we conclude that MuRF1 conforms to the canonical model of TRIM fold and that the COS-box does not constitute an independent C-terminal domain in MuRF1.

## Results

### Cross-linking analysis suggests that MuRF1-HD is an antiparallel dimer

**Fig. 2:**
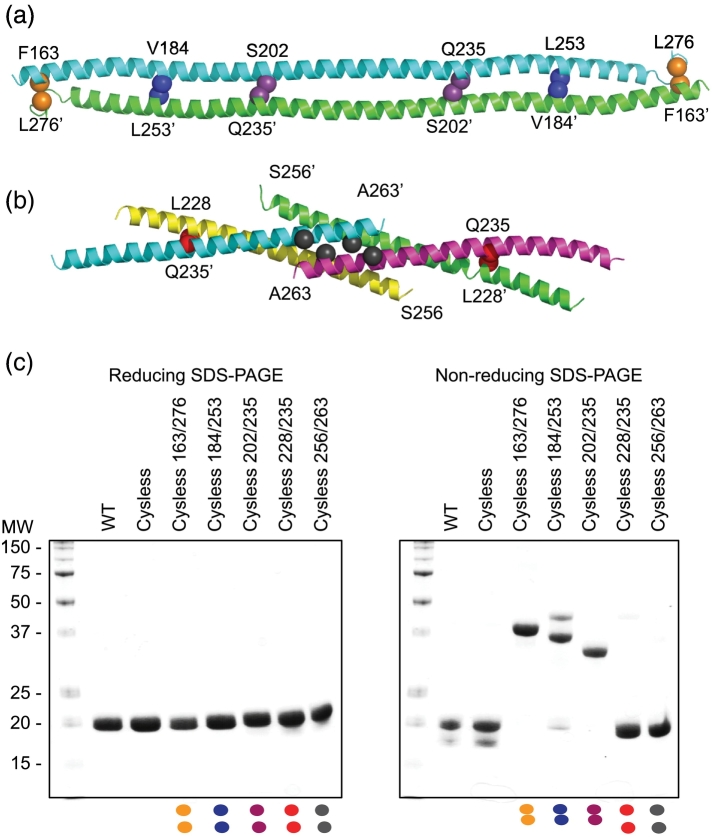
Disulfide cross-linking of the MuRF1-HD dimer. (A) Polyalanine homology model of helix H1 in MuRF1-HD using the antiparallel TRIM25 dimer as template. Positions selected for cysteine substitutions are indicated by spheres and labeled. (B) Crystal structure of the central fraction of helix H1 from MuRF1 as two intertwined parallel dimers (PDB: 4M3L). Cysteine substitutions are denoted by spheres as in panel A. (C) SDS-PAGE profiles of purified MuRF1-HD cysteine mutants analyzed under reducing (left) and non-reducing (right) conditions. Double cysteine mutants (made in a cysteine-less C173S, C293S background) based on the antiparallel model migrated as dimers under non-reducing conditions, whereas mutants based on the parallel structure migrated as monomers (color coded dots match color scheme in panels A and B).

MuRF1 is composed of a RING domain in N-terminal position, a MuRF-specific helical motif, a B-box type II (B), an HD that includes the COS-box sequence motif, and a disordered C-terminal acidic tail (AT) (Fig. 1). The HD of MuRF1 (MuRF1-HD) is predicted to consist of a long, primary helix (H1) and two shorter helices (H2 and H3) [18], resembling the secondary structure content of HD domains in structurally characterized TRIMs. MuRF1-HD has been shown to form dimers in solution by multi-angle laser light scattering coupled to size exclusion fractionation, independently of the presence or absence of the AT [15], [23]. To establish the assembly mode of MuRF1, recombinant MuRF1-HD was produced in the current work as a stable, soluble protein product of high purity. To test whether MuRF1-HD associated in an antiparallel fashion, we applied a “zero-length” disulfide crosslinking strategy previously described for TRIM25 and TRIM5α [8]. Briefly, we introduced the mutation pairs F163C/L276C, V184C/L253C and S202C/Q235C in a cysteine-null variant of MuRF1-HD, generated by mutagenesis of two native cysteines (C173S and C293S) (all mutations in the study, and their sampling of the HD domain, are shown in Fig. 1). Samples of the cysteine-null and double-cysteine MuRF1-HD mutants behaved similarly to the wild-type sample during production and purification, suggesting that mutagenesis had not introduced significant alterations in the fold. The double-cysteine substitutions introduced in MuRF1-HD correspond to residue positions that are in close proximity in the crystal structure of TRIM25-HD (Fig. 2A). The cysteine pairs could be expected to spontaneously oxidize into disulfide bonds if the MuRF1-HD dimer adopted an antiparallel arrangement. Upon cross-linking by spontaneous oxidation, we found that the double mutants F163C/L276C, V184C/L253C and S202C/Q235C predominantly migrated as dimers under non-reducing conditions but migrated as monomers on reducing SDS-PAGE similar to the wild-type and cysteine-less controls (Fig. 2C). We next performed the same analysis on the mutants L228C/Q235C and S256C/A263C, wherein the cysteine pairs were designed based on the truncated coiled-coil structure of MuRF1 [18] (Fig. 2B). These double-cysteine mutants migrated as monomers in SDS-PAGE under both reducing and non-reducing conditions (Fig. 2C), indicating that the cysteine substitutions were too far away from each other to form disulfides. Therefore, we concluded that the MuRF1-HD dimer adopted an antiparallel arrangement (Fig. 2A).

### PELDOR distance measurements confirm the antiparallel TRIM fold of MuRF1-HD

To obtain a more detailed characterization of the MuRF1-HD fold, we sampled intermolecular distances across protomers in the dimer using EPR and applying the PELDOR protocol. The PELDOR experiment measures the dipolar coupling between the unpaired electrons in spin-labels introduced in proteins. The data can be interpreted as a distance and a distance distribution, if the data are of sufficient quality. The application of the data provided by PELDOR to the interpretation of the underlying protein structure is often complicated by the conformation and dynamics of the flexible spin labels. The dynamics of the latter is not necessarily homogeneous. Spin labels can form spatial subpopulations, possibly due to steric hindrance effects or unspecific interactions with the protein surface, which yield split distance distributions of complex appearance. In such cases, it is the modal distance and the shape of the distance distribution that are informative, with an experimental distance error factor not being directly deductible from the distributions themselves. When a reliable atomic structure of the protein under study exists, a complex distance distribution can be studied through the modeling and molecular dynamics simulation of spin labels introduced into the structure *in silico*. Matching the simulated subpopulations of spin labels to the features of EPR distance distributions provides an insight into the nature of their various peaks and permits identifying possible outlier features. In the absence of an atomic structure, the modeling of spin labels can be performed using homology models with caution.

**Fig. 3:**
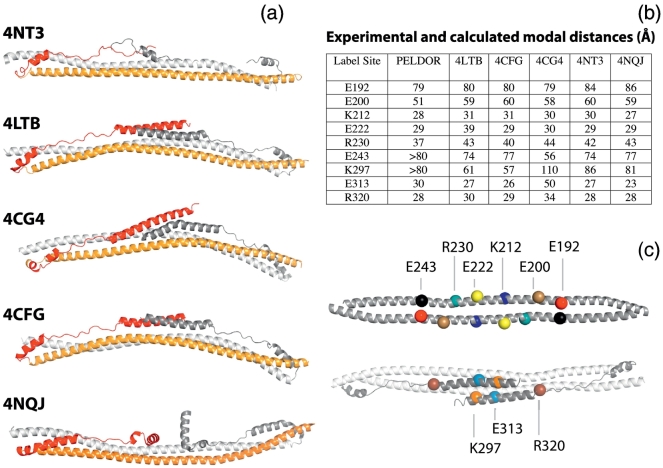
Distance measurements and label positions. (A) Crystal structures of HDs from TRIMs with PDB codes as indicated (the B-box domain of 4TN3 is removed to ease comparison). The structures were used for the threading of the MuRF1 sequence and used as homology models. (B) Table showing the modal distances (in Å) for each spin-label position, derived from PELDOR data and from homology models. PELDOR data for E243 and K297 are insufficient to define an accurate distance but do allow the conclusion that the distances are greater than 80 Å. (C) Cartoon representation of MurF1 model based on structure 4LTB (TRIM25) with label positions highlighted in color and by spheres at the requisite Cα carbon.

**Fig. 4:**
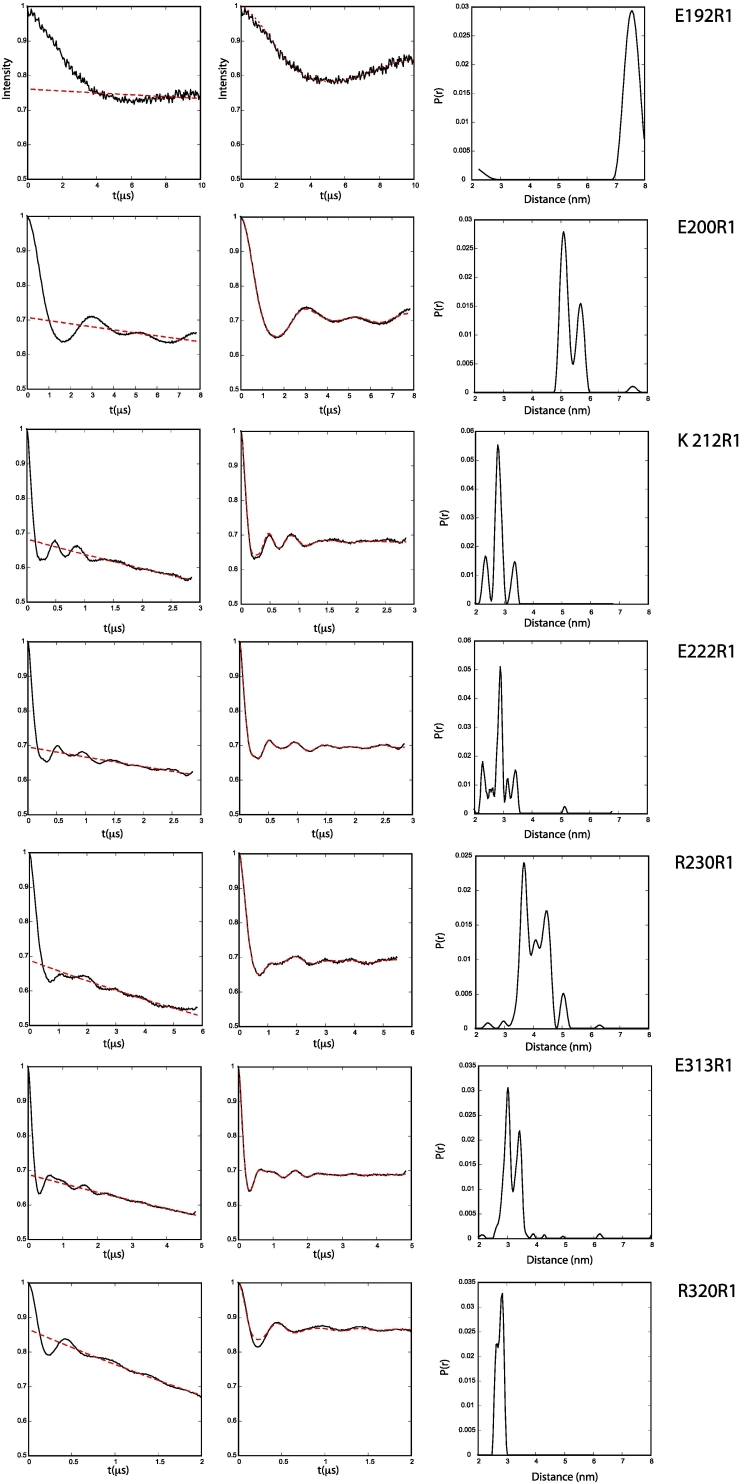
PELDOR data. Raw and processed PELDOR measured from MuRF1-HD constructs. Column 1 shows raw data with the dashed red lines indicating the function used for correcting the intermolecular contributions, column 2 shows background corrected data, with the dashed red lines indicating the fit for the background-corrected data from Tikhonov regularization, and column 3 shows the distance distribution derived by Tikhonov regularization. Data for E243 and K297 shown in Fig. S4. Due to a less than complete oscillation in the Peldor data for E192, background validation results are shown in Fig. S5. PELDOR data. Raw and processed PELDOR measured from MuRF1-HD constructs. Column 1 shows raw data with the dashed red lines indicating the function used for correcting the intermolecular contributions, column 2 shows background corrected data, with the dashed red lines indicating the fit for the background-corrected data from Tikhonov regularization, and column 3 shows the distance distribution derived by Tikhonov regularization. Data for E243 and K297 shown in Fig. S4. Due to a less than complete oscillation in the Peldor data for E192, background validation results are shown in Fig. S5.

In order to study MuRF1-HD using EPR, nine residues in the cysteine-null MuRF1-HD segment were exchanged for cysteines using site-directed mutagenesis: E192C, E200C, K212C, E222C, R230C, E243C, K297C, E313C and R320C (Fig. 1). The residue exchanges were selected as to sample the length of the protein, within the limitations of the EPR distance measurement, while preserving putative coiled-coil interactions. All resulting MuRF1-HD variants could be expressed as soluble, stable protein products. Proteins were subsequently modified by reaction of the exposed cysteine groups with the spin label MTSSL (below indicated as R1), and modal distances (Fig. 3B) and distributions (Fig. 4) between labels were derived from PELDOR data. The data derived from PELDOR were of mixed quality (Fig. 4). Data for E200R1, K212R1, E222R1 and R230R1 on the MuRF1-H1, as well as E313R1 and R320R1 on the COS-box, contained two or more full oscillations allowing a modal distance and distribution to be measured. In addition, the single oscillation visible in the raw PELDOR data for E192R1 allowed a modal distance to be measured at this site. E243R1 on helix-H1 as well as K297R1 on the COS-box gave less than one full oscillation. This does not allow a distance estimation to be made with any great accuracy but does allow us to conclude that the label-label distances are greater than 8 nm. The raw data are shown in Fig. S4.

Next, we analyzed the MuRF1 inter-label distances recorded by EPR by investigating their compatibility with the fold of other TRIMs characterized structurally to date. For this, homology models of MuRF1-HD were calculated with Modeller [24] based on the available crystal structures. Specifically, the crystal structures for the helical regions of TRIM5α (4TN3 [25]), TRIM20 (4CG4 [26]), TRIM69 (4NQJ [27]) and TRIM25 (4LTB [28] and 4CFG [29]) were used as individual model templates (Fig. 3A). The spin label R1 was then computationally introduced in each of the resulting MuRF1-HD models using MTSL Wizard [30] and the modal distance calculated and compared to experimental PELDOR values (Fig. 3B). The modal distances from modeled and experimental data were comparable for all sites on the long helix H1 for which oscillations could be recorded (E192R1, E200R1, K212R1, E222R1 and R230R1). Oscillation-free PELDOR data for E243R1 gave a distance estimate that was close to all the MuRF1-HD homology models, except that calculated from 4CG4. PELDOR is an especially effective technique for determining whether a homodimeric coiled-coil is in a parallel or antiparallel conformation. Labels in antiparallel coiled-coils show a wide range of distances, but in parallel conformations, the distances between labels remain small and similar, irrespective of position in the sequence [31]. Effectively, the experimentally measured distances from MuRF1-HD samples were shorter for sites located within the central part of helix H1, but became longer for sites at either terminus of the helix (Fig. 3B), as expected for an antiparallel packing of helix H1 in the dimer. In conclusion, the analysis of the distances in MuRF1-HD demonstrated an antiparallel association of the monomers and a packing of the long H1 helices in the dimer that resembles the fold of other TRIM proteins.

### The COS-box of MuRF1 does not form an alpha-helical hairpin

In most of the previously published TRIM protein structures (Fig. 3A), the polypeptide chain C-terminal to the dimerization domain is seen to be folded back over the dimerization domain and interacts in the H3 region forming a four-helical bundle with the center of helix H1. However, there is some variability in the degree of order in this region across the different structures. The experimental distances derived from E313R1 and E320R1 are relatively short and compare well with the modeled distances seen in 4LTB, 4CFG and 4TN3, implying a similarity in structure. The distance measured for K297R1 is similar only to 4TN3 and 4NQJ. Overall, the similarity between the experimental distances for H3 (K297R1, E313R1 and R320R1) and those modeled using 4LTB is quite striking, generally being compatible with an antiparallel packing of H3 against H1 with a central crossing point around residue 316. The narrow distance distribution for E313R1 and R320R1 suggests that the antiparallel arrangement of H3 is well ordered and stable (at least under the conditions of the PELDOR experiment).

To further validate the conformation of the COS-box of MuRF1, we performed a triangulation experiment by determining distances using a double label strategy with labels placed at E192R1 and R320R1 simultaneously. Because measurement in systems containing more than two spin labels can be problematic [32], the data in this case were gathered such that no oscillation between E192R1 and its symmetry partner was observed, the distances between the labels being too long to measure. A distance distribution between R320R1 and itself was observed and was consistent with that measured alone. Two additional significant peaks were observed that corresponded to interactions between E192R1 and R320R1 (Fig. S2). The distances observed with the double-labeled sample were consistent with the calculated MuRF1 model based on the PDB structure 4LTB and are a useful confirmation that the C-terminal helix H3 was indeed stacked over the center of the dimerization domain.

## Conclusion

The TRIM fold appears to be an anisometric, rod-like fold, where functional domains are organized onto a central antiparallel coiled-coil of approximately 18 nm length. Only a few examples of partial structures from this family are known to date, the first structures from the central TRIM rod domain being reported in 2014 [6], [8], [10]. Out of the 11 TRIM sub-classes [4], only HDs from members of class C-IV (TRIM5, TRIM25 and TRIM69) and one unclassified protein (TRIM20) have been structurally characterized so far, with possible variability within the fold yet to be revealed. EPR is a particularly effective technique for measuring distances within a homodimeric rod-like fold, especially to distinguish parallel and antiparallel chain arrangements.

Here, we have studied the HD dimerization domain of MuRF1 (TRIM63), a small TRIM protein of class C-II that lacks C-terminal specific domains but contains a conserved sequence motif, the COS-box. Earlier data on MuRF1 [18] as well as on the COS-box of MID1 (TRIM18 in class C-I) had led to an expectation of fold differences in these proteins. We started this study with three pieces of structural information on MuRF1; one being the proposed structure of its COS-box [17], [18], another was a crystal structure of a fraction of its HD dimerization domain [18] and the third was the homology to other TRIM proteins. The data presented here permit discriminating between the somewhat contradictory structural information. The EPR distances measured in this work and the cross-linking studies that used introduced disulfide bridges, are compatible with an antiparallel arrangement of MuRF1-HD and with a regular structure of the COS-box that emulates the corresponding fold section in other TRIMs. In summary, we conclude that COS-box containing proteins in the classes C-I, C-II and C-III of the TRIM family exhibit a canonical TRIM fold.

## Methods

### Cloning

The HD of MuRF1, MuRF1-HD (residues 155–328; UnitProtKB Q969Q1), was cloned into a modified pET28a vector (Novagen) that adds a His_6_-tag and a SUMO domain N-terminal to the inserted gene. A cysteine-null MuRF1-HD sample was generated by exchanging native cysteine residues (C173 and C293) into serines using QuikChange® (Agilent). For cross-linking and EPR analyses, cysteines were introduced into the cysteine-nullMuRF1-HD at positions F163, V184, E192, E200, S202, K212, E222, L228, R230, Q235, E243, L253, S256, A263, L276, K297, E313 and R320, using the same mutagenesis protocol. All expression plasmids were confirmed by sequencing.

### Protein production

All proteins were expressed in *Escherichia coli* BL21 (DE3) (Agilent). Cells were cultivated in Luria-Bertani medium supplemented with 50 μg/mL kanamycin at 37 °C to an OD_600_ = 0.8. Protein expression was induced using 0.2 mM IPTG, and cultures were grown further for 3.5 h at 37 °C. Cells were harvested by centrifugation and lysed by sonication in 50 mM Tris–HCl (pH 8.0), 500 mM NaCl, 5% (v/v) glycerol and 5 mM β-mercaptoethanol in the presence of an EDTA-free protease inhibitor cocktail (Roche). The lysate was clarified by centrifugation and subsequent filtration. The purification of all proteins from supernatants followed Ni^2+^-chelating affinity chromatography. The His_6_-SUMO tag was cleaved with His-Ulp1 protease in overnight dialysis [30 mM Tris–HCl (pH 8.0), 100 mM NaCl, 5% (v/v) glycerol, 5 mM β-mercaptoethanol] at 5–7 °C and the proteolyzed mixture subjected to subtractive Ni^2+^-NTA chromatography.

### Cysteine cross-linking

Double-cysteine mutant proteins were reduced by dilution to 20 μM in reducing buffer [50 mM Tris–HCl (pH 8.0), 100 mM NaCl, 20 mM β-mercaptoethanol] and then dialyzed overnight at 4 °C into the same buffer containing no reducing agent. Aliquots were then mixed with the same volume of 2 × SDS-PAGE sample buffer containing either 0 (nonreducing) or 1 M β-mercaptoethanol (reducing), incubated for 5 min at 99 °C in a dry bath and immediately analyzed by SDS-PAGE with Coomassie-blue staining.

### EPR sample preparation

Cysteine residues were reduced by incubation in 5 mM DTT for 2 h at room temperature. DTT was removed with size exclusion chromatography using a 10/300 Superose S12 column (GE Healthcare) in labeling buffer [20 mM Hepes (pH 6.8), 100 mM NaCl). MuRF1 constructs were then labeled by incubation with a 10-fold molar excess of MTSSL for 2 h at room temperature. Excess label was removed by dialysis in 20 mM Hepes (pH 8.0) and 100 mM NaCl at 5–7 °C overnight.

PELDOR samples were buffer-exchanged into 2 × concentrated dialysis buffer, prepared with D_2_O, using centrifugal ultrafiltration. Buffer exchanged samples were concentrated to a final concentration of 100 to 200 μM MuRF1 dimer in a volume of 50 μL and mixed in a 1:1 (v/v) ratio with D_8_-glycerol. For PELDOR measurements, 75 μL of each sample was transferred to a quartz tube and flash frozen to form a glass. MuRF1 labeling efficiency was assessed by Continuous Wave (CW) EPR using an amino-TEMPO standard as reference (Fig. S6).

### Distance measurement

PELDOR experiments were performed using a Bruker ELEXSYS E580 spectrometer operating at Q-band with a cylindrical resonator ER 5106QT-2w and a Bruker 400-U second microwave source unit. All measurements were taken at a temperature of 50 K with an over coupled resonator giving a *Q* factor of ~ 250–300. The video bandwidth was 20 MHz. The spectrometer was equipped with a cryogen-free variable temperature cryostat (cryogenic limited) operating in the 1.5–300 K temperature range. Pulses were amplified using a pulsed traveling wave tube amplifier with a nominal power output of 150 W. The four-pulse, dead-time free PELDOR sequence was used, with the pump pulse frequency positioned at the maxima of the nitroxide spectrum. The frequency of the observer pulses was incremented by 80 MHz relative to the pump position. The observer sequence used a 32-ns π-pulse; the pump π-pulse was typically 16 ns. The experiment repetition time was 4 ms, and the number of shots at each time point was 50. The number of time points and the number of scans used were varied for each sample, but sufficient data were collected to obtain an adequate signal-to-noise ratio. Data were analyzed using the DeerAnalysis 2013 software package [33]. The raw data were corrected for background echo decay using a homogeneous three-dimensional spin distribution. The starting time for the background fit was optimized to give the best-fit Pake pattern in the Fourier-transformed data and the lowest root-mean-square deviation background fit (Fig. S3). Tikhonov regularization was used to derive distance distributions *P*(*r*).

### Homology modeling

Pairwise sequence alignments were performed using the EMBOSS Needle server [34] using a gap penalty 10, a gap extension penalty of 5.0, an end gap penalty of 10 and an end gap extension penalty of 1. The higher gap extension penalty when compared to the default value of 0.5 was used to maintain the alignment of the heptad repeats in the sequence of the coiled coil domain. The automodel feature of Modeller [24] was used to model MuRF1-HD (residues 161–324) using protein structure templates available at the Protein Data Bank: 4LTB, TRIM25; 4CFG, TRIM25; 4CG4, TRIM20; 4TN3, TRIM5α; 4NQJ, TRIM69 (sequence alignment shown in Fig. S1). Distance distributions, *P*(*r*), were calculated from the resulting MuRF1 homology models using MTSSL-Wizard [30] and compared to the PELDOR experimental distance distributions. (See Fig. S5.)

The following are the supplementary materials related to this article.

Fig. S1 Alignment of TRIM-HD sequences. Heptad positions (adapted from Ref. [8]) for H1 are indicated and position *d* residues are boxed in red. For each TRIM protein, helical sequences have been colored (H1, orange; H2, blue; H3, green), according to features observed in the respective PDB structures for TRIM25, TRIM69, TRIM20 and TRIM5α. For TRIM63, helices have been colored according to secondary structure prediction from Jpred (http://www.compbio.dundee.ac.uk/jpred). Fig. S1

Fig. S2 EPR triangulation experiment to determine the position of the COS-box relative to the coiled-coil. (A) The raw PELDOR data with background correction function. (B) Background corrected data with fit to the distance distribution, and (C) the distance distribution with background validation, for construct labeled at residues 192 and 320. The lower and upper error bounds (two times standard deviation) are displayed as gray error bars. (D) The Pake pattern derived from the background corrected data with fit. (E) The L-curve used to determine optimum fit characteristics. (F) A comparison between the experimentally determined distance distribution (black) and the distributions calculated from the model based on PDB 4LTB, with spin labels added using MtsslWizard [30]. The shorter distance corresponds to a distance of 2.7 nm (residues 320–320), the two main longer distances correspond to 4.2 and 4.9 nm (residues 320–192), and the shoulder at approximately 3.6 nm may either be an artfact of the multiple labeling experiment or a distance associated with a minor conformation of the spin-label position. The lower figure shows the positions of the spin labels on the modeled structure as two orthogonal views. Fig. S2

Fig. S3 PELDOR data. Tikhonov regularization of the PELDOR data. Column 1 shows the Pake patterns, and column 2 shows the L-curves for the data shown in Fig 4. Regularization parameters derived from the L-curves are as follows: E192, 158; E200, 32, K212, 5, E222, 1, R230; 15, E313, 5; and R320, 2. Fig. S3

Fig. S4 PELDOR data from label positions E243R1 and K297R1. The PELDOR data derived from these positions gave data that are insufficient to derive an accurate distance but are indicative of label distances being in excess of 8 nm. Fig. S4

Fig. S5 Results of the background verification using DeerAnalysis on sample E192R1. The lower and upper error bounds (two times standard deviation) are displayed as gray error bars. Mean distance, 76 Å; Std, 2.4 Å. Fig. S5

Fig. S6 CW spectra for samples used in PELDOR distance estimations. CW spectra were taken during preparation and labeling of samples to estimate labeling efficiency. Comparison to a sample of free R1 label was used with double integration to estimate the concentration of spin label which, in concert with an estimation of protein concentration taken from UV measurement and predicted absorption coefficient, was used to estimate the efficiency of labeling. Labeling efficiency was estimated to be 90% or greater for all samples, except that for R320 which appeared to be lower judging from the PELDOR oscillation depth. Some samples contained small amounts of free label, which would distort the calculation, but at the levels observed, had little effect on the PELDOR experiment. PELDOR experiments resulted in oscillation depths of 0.3 and 0.15 giving sufficient signal to determine distances, which are otherwise unaffected by labeling efficiency. Fig. S6
