# Supplementary material for: Exploration of the TRIM Fold of MuRF1 Using EPR Reveals a Canonical Antiparallel Structure and Extended COS-Box
Source: J Mol Biol. 2019 Jul 12;431(15):2900–9. doi: 10.1016/j.jmb.2019.05.025 (PMC6599887; doi:10.1016/j.jmb.2019.05.025)
Supplement: Fig. S1 — Alignment of TRIM-HD sequences. Heptad positions (adapted from Ref. [8]) for H1 are indicated and position d residues are boxed in red. For each TRIM protein, helical sequences have been colored (H1, orange; H2, blue; H3, green), according to features observed in the respective PDB structures for TRIM25, TRIM69, TRIM20 and TRIM5α. For TRIM63, helices have been colored according to secondary structure prediction from Jpred (http://www.compbio.dundee.ac.uk/jpred). [file mmc1.pdf]

```
                                          defgabcdefgabcdefgabcdefgabcdefghijkabcdefghijkabcdefghijkabcdefghijk
TRIM63              ----------LQSVFQGQKTELNNCISMLVAGNDRVQTIITQLEDSRRVTKENSHQVKEELSQKFDTLYAILDEKKSELLQRITQEQEKK
TRIM25:4LTB/4CFG    --------ASLSQASADLEATLRHKLTVMYSQINGASRALDDVRNRQQDVRMTANRKVEQLQQEYTEMKALLDASETTSTRKIKEEEKRV
TRIM69:4NQJ         SVGQSKEFLQISDAVHFFMEELAIQQGQLETTLKELQTLRNMQKEAIAAHKENKLHLQQHVSMEFLKLHQFLHSKEKDILTELREEGKAL
TRIM20:4CG4         -----------SEVALEHKKKIQKQLEHLKKLRKSGEEQRSYGEEKAVSFLKQTEALKQRVQRKLEQVYYFLEQQEHFFVASLEDVGQMV
TRIM5a:4TN3         ----------MEEVAQEYHVKLQTALEMLRQKQQEAEKLEADIREEKASWKIQIDYDKTNVSADFEQLREILDWEESNELQNLEKEEEDI

                    abcdefgabcdefgabcdefgabcdefgabcd
TRIM63              LSFIEALIQQYQEQLDKSTKLVETAIQSLDEPGGATFLLTAKQLIKSIVEASKGCQ--------LGKTEQGFENMDFFTLDLEHIADALRAID--FGTD
TRIM25:4LTB/4CFG    NSKFDTIYQILLKKKSEIQTLKEEIEQSLTKRDEFEFLEKASKLRGISTKPVYIPE----VELNHKLIKGIHQSTIDLKNELKQCIGRLQELTPSSGDP
TRIM69:4NQJ         NEEMELNLSQLQEQCLLAKDMLVSIQAKTEQQNSFDFLKDITTLLHSLEQGMKVLA---TRELISRKLNLGQ-----YKGPIQYMVWREMQDTLCPG--
TRIM20:4CG4         GQIRKAYDTRVSQDIALLDALIGELEAKE-CQSEWELLQDIGDILHRA-KTVPVPEKWTTPQEIKQKIQLLHQKSEFVEKSTKYFSETLRSEMEMFNVP
TRIM5a:4TN3         LKSLTKSETEMVQQTQYMRELISELEHRL-QGSMMDLLQGVDGIIKRI-ENMTLKK----PKTFHKNQRRVFRAPD-LKGMLDMFRDAAAEESPVL---
```
