# Supplementary figures and images for: Exploration of the TRIM Fold of MuRF1 Using EPR Reveals a Canonical Antiparallel Structure and Extended COS-Box
Source: J Mol Biol. 2019 Jul 12;431(15):2900–9. doi: 10.1016/j.jmb.2019.05.025 (PMC6599887; doi:10.1016/j.jmb.2019.05.025)

# E192R1/R320R1

**A**

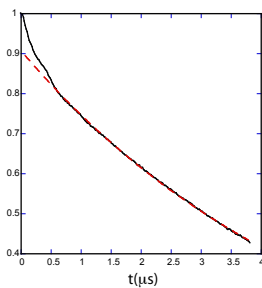

**B**

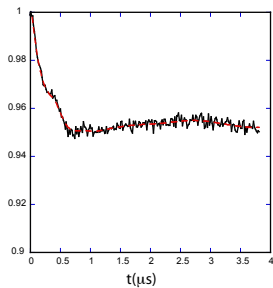

**C**

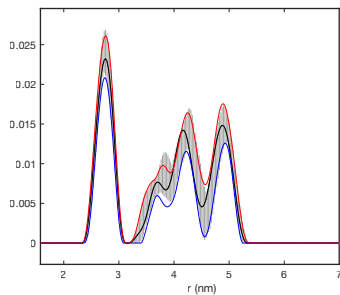

**D**

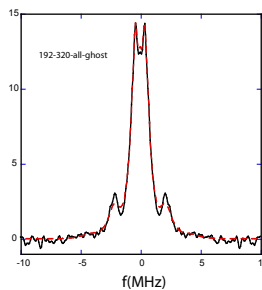

**E**

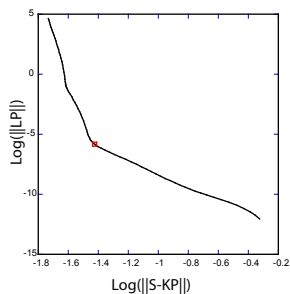

**F**

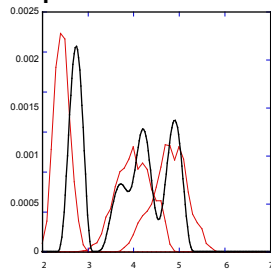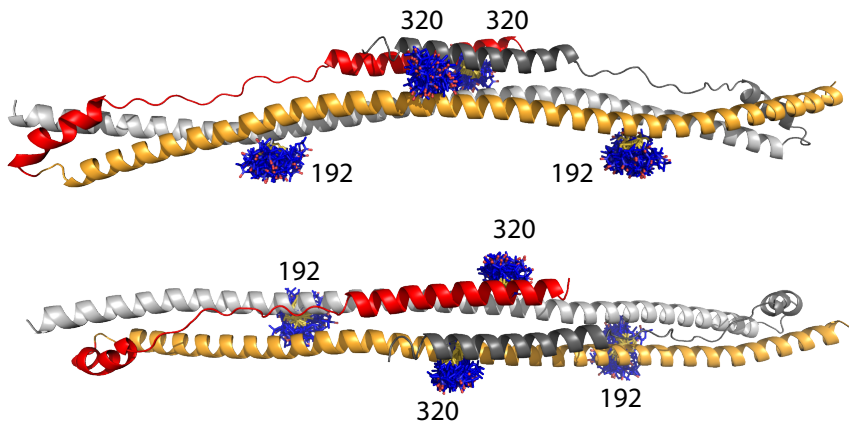

Supplement: Fig. S2 — EPR triangulation experiment to determine the position of the COS-box relative to the coiled-coil. (A) The raw PELDOR data with background correction function. (B) Background corrected data with fit to the distance distribution, and (C) the distance distribution with background validation, for construct labeled at residues 192 and 320. The lower and upper error bounds (two times standard deviation) are displayed as gray error bars. (D) The Pake pattern derived from the background corrected data with fit. (E) The L-curve used to determine optimum fit characteristics. (F) A comparison between the experimentally determined distance distribution (black) and the distributions calculated from the model based on PDB 4LTB, with spin labels added using MtsslWizard [30]. The shorter distance corresponds to a distance of 2.7 nm (residues 320–320), the two main longer distances correspond to 4.2 and 4.9 nm (residues 320–192), and the shoulder at approximately 3.6 nm may either be an artfact of the multiple labeling experiment or a distance associated with a minor conformation of the spin-label position. The lower figure shows the positions of the spin labels on the modeled structure as two orthogonal views. [file mmc2.pdf]

E192R1

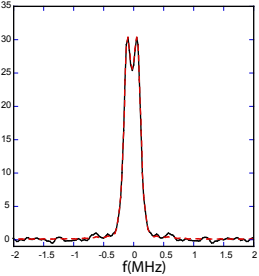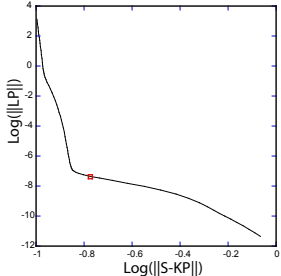

E200R1

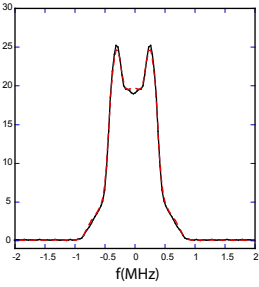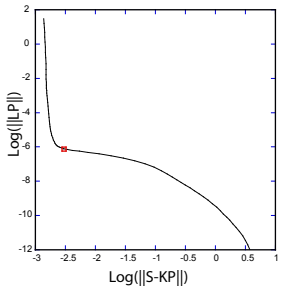

K212R1

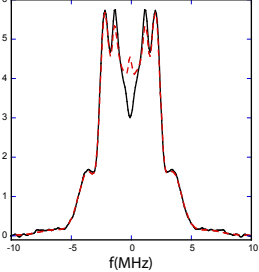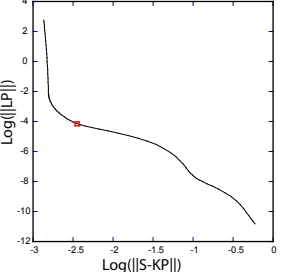

E222R1

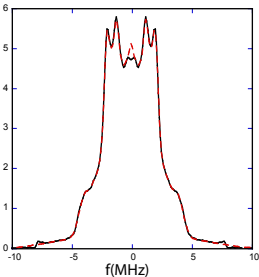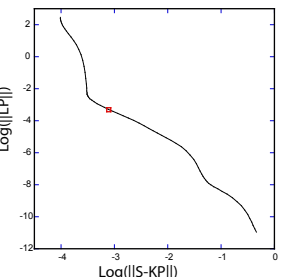

R230R1

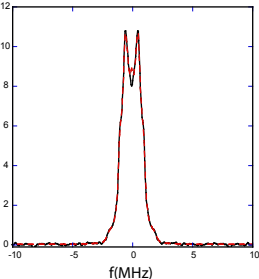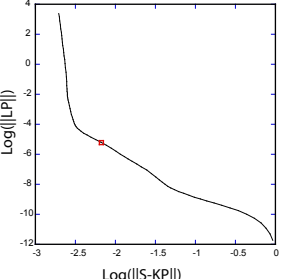

E313R1

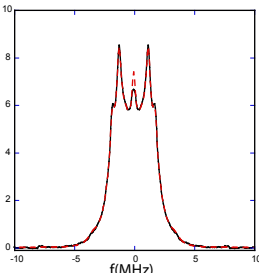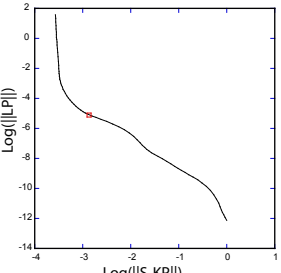

R320R1

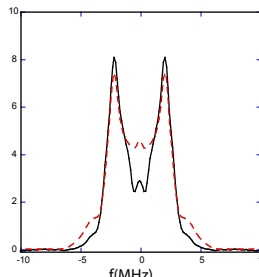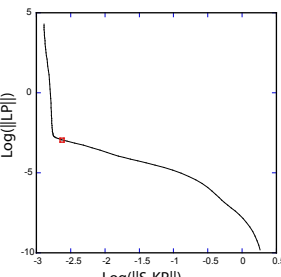

Supplement: Fig. S3 — PELDOR data. Tikhonov regularization of the PELDOR data. Column 1 shows the Pake patterns, and column 2 shows the L-curves for the data shown in Fig 4. Regularization parameters derived from the L-curves are as follows: E192, 158; E200, 32, K212, 5, E222, 1, R230; 15, E313, 5; and R320, 2. [file mmc3.pdf]

E243R1

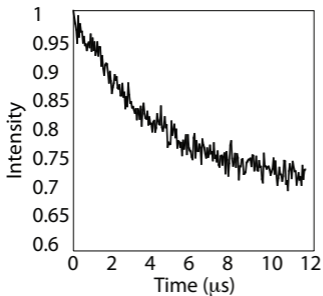

K297R1

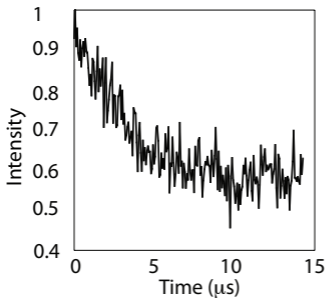

Supplement: Fig. S4 — PELDOR data from label positions E243R1 and K297R1. The PELDOR data derived from these positions gave data that are insufficient to derive an accurate distance but are indicative of label distances being in excess of 8 nm. [file mmc4.pdf]

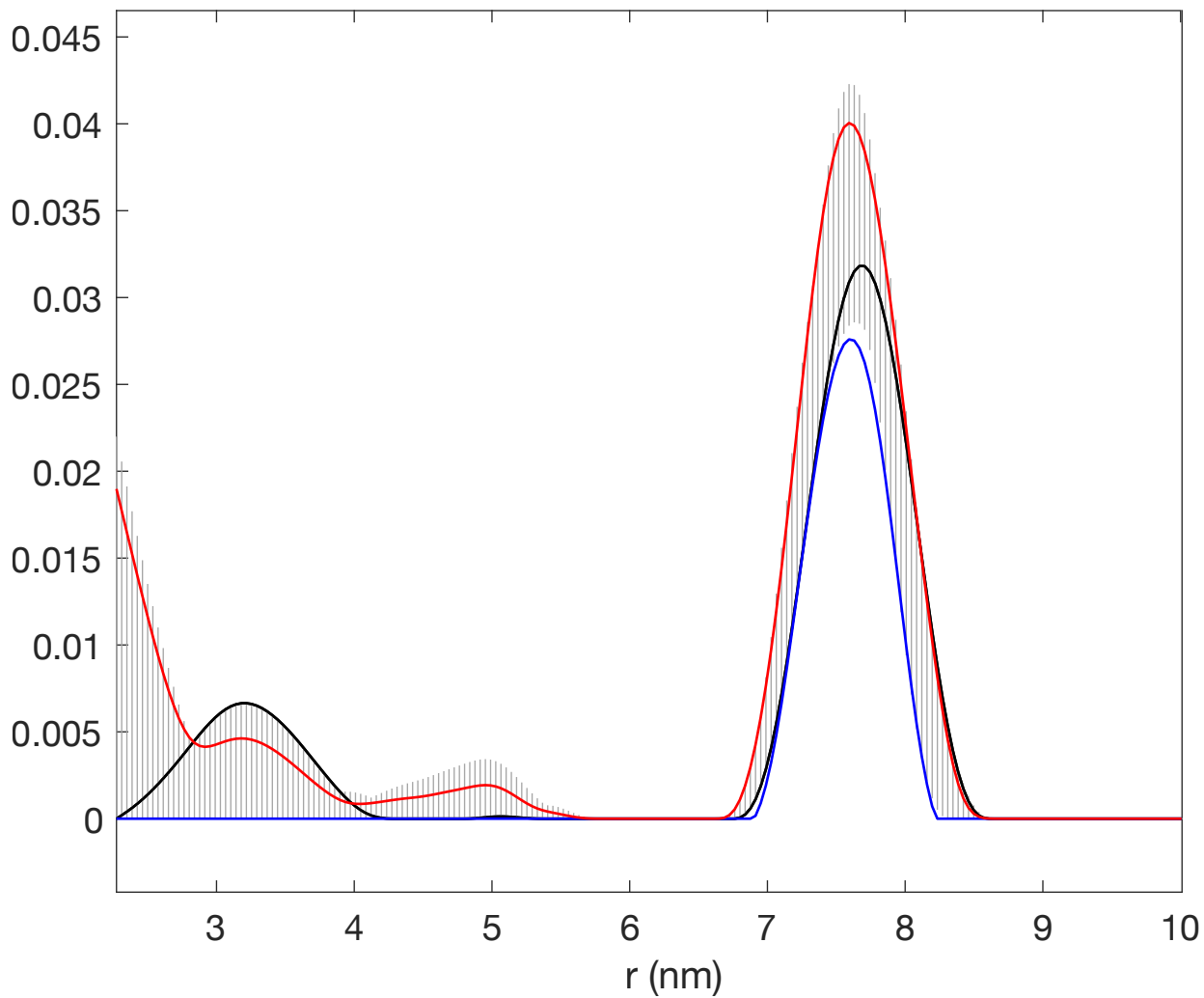

Supplement: Fig. S5 — Results of the background verification using DeerAnalysis on sample E192R1. The lower and upper error bounds (two times standard deviation) are displayed as gray error bars. Mean distance, 76 Å; Std, 2.4 Å. [file mmc5.pdf]

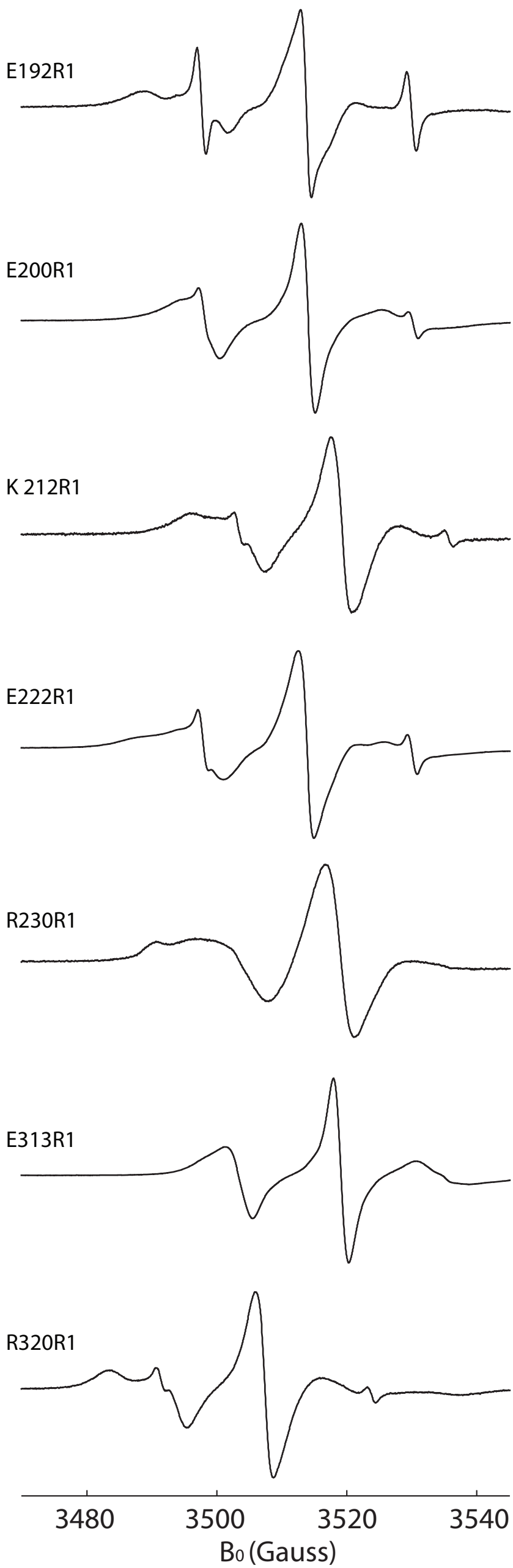

Supplement: Fig. S6 — CW spectra for samples used in PELDOR distance estimations. CW spectra were taken during preparation and labeling of samples to estimate labeling efficiency. Comparison to a sample of free R1 label was used with double integration to estimate the concentration of spin label which, in concert with an estimation of protein concentration taken from UV measurement and predicted absorption coefficient, was used to estimate the efficiency of labeling. Labeling efficiency was estimated to be 90% or greater for all samples, except that for R320 which appeared to be lower judging from the PELDOR oscillation depth. Some samples contained small amounts of free label, which would distort the calculation, but at the levels observed, had little effect on the PELDOR experiment. PELDOR experiments resulted in oscillation depths of 0.3 and 0.15 giving sufficient signal to determine distances, which are otherwise unaffected by labeling efficiency. [file mmc6.pdf]
